# Hybrid CNN with angular margin supervision for robust face identification and verification

**DOI:** 10.3389/frai.2026.1816796

**Published:** 2026-07-27

**Authors:** Andisani Nemavhola, Colin Chibaya, Serestina Viriri

**Affiliations:** 1 School of Consumer Intelligence and Information Systems, University of Johannesburg, Johannesburg, South Africa; 2 School of Natural and Applied Sciences, Sol Plaatje University, Kimberley, South Africa; 3 School of Mathematics, Statistics and Computer Science, University of KwaZulu-Natal, Durban, South Africa

**Keywords:** angular margin learning, ArcFace loss, biometric verification, face recognition, hybrid convolutional neural network, Softmax loss

## Abstract

Face recognition systems are widely used in surveillance, biometric authentication, access control, and digital identity verification; however, supervision sensitivity, evaluation stability, and performance consistency across datasets remain insufficiently understood. This study investigates the behavior of convolutional, transformer-based, and hybrid face recognition architectures under both Softmax and ArcFace supervision using five-fold subject-disjoint cross-validation on the Labeled Faces in the Wild (LFW) and FAGEv2 datasets. ResNet50, MobileNetV3, DeiT-Small, and a Hybrid multi-branch architecture integrating complementary convolutional and transformer feature representations were evaluated using Top-1 identification accuracy, Area Under the ROC Curve (AUC), Equal Error Rate (EER), computational complexity, and fold-level statistical analysis. Experimental results revealed substantial supervision sensitivity across architectures and datasets. On the LFW dataset, Hybrid-Softmax achieved the highest Top-1 identification accuracy (62.4%), while DeiT-Small-Softmax achieved the strongest verification performance with an AUC of 0.905 and EER of 0.159. On the FAGEv2 dataset, Hybrid-Softmax and DeiT-Small-Softmax achieved the highest identification accuracy (38.0%), while Hybrid-Softmax achieved the strongest verification performance with an AUC of 0.825 and EER of 0.251. Fold-level analyses demonstrated that the effect of ArcFace supervision varied across architectures and datasets, with consistent improvements observed for some convolutional architectures but not for transformer-based or hybrid models. Cross-dataset evaluation further revealed changes in model ranking and supervision behavior, indicating that comparative performance is strongly influenced by dataset characteristics and evaluation conditions. The findings demonstrate that additive angular margin supervision does not universally outperform conventional Softmax optimization and highlight the importance of multi-dataset benchmarking, fold-level evaluation, and supervision sensitivity analysis for robust and reproducible face recognition benchmarking.

## 1 Introduction

Face recognition has become one of the most widely deployed biometric technologies, supporting applications in surveillance, border control, mobile authentication, financial security, and digital identity verification (Nemavhola et al., 2025a,b). Recent advances in deep learning have significantly improved face identification and verification performance through convolutional neural networks (CNNs), transformer-based architectures, and margin-supervised embedding learning strategies. Despite these improvements, concerns regarding evaluation reliability, reproducibility, and cross-dataset generalization have become increasingly important in contemporary biometric research.

Convolutional neural networks remain dominant in practical face recognition systems due to their strong local feature extraction capability and computational efficiency (Ranjan et al., 2019). Architectures such as ResNet50 provide deep hierarchical structural representations through residual learning, while lightweight models such as MobileNetV3 employ depthwise separable convolutions to reduce computational complexity for edge deployment scenarios. More recently, transformer-based architectures have demonstrated strong global contextual modeling capability through self-attention mechanisms (Palanisamy et al., 2025). However, transformer models often require larger-scale training diversity and increased computational resources to achieve stable generalization performance.

To improve discriminative representation learning, modern face recognition systems increasingly employ angular marginbased supervision strategies such as ArcFace. By enforcing hyperspherical embedding constraints, additive angular margin losses improve intra-class compactness and inter-class separability in embedding space (Fuad et al., 2021). Although ArcFace has demonstrated strong performance on several large-scale benchmarks, emerging evidence suggests that the effectiveness of margin-based supervision may depend on dataset structure, architectural design, and evaluation conditions, particularly under constrained or cross-dataset scenarios.

At the same time, hybrid architectures combining convolutional and transformer-based feature extraction have emerged as a promising direction for representation learning in computer vision tasks (Long, 2024). Such approaches aim to exploit complementary strengths: convolutional networks capture fine-grained local texture patterns, while transformer or deeper structural modules model global dependencies and contextual relationships. Nevertheless, the statistical reliability and cross-dataset stability of hybrid face recognition architectures remain insufficiently explored.

Another major limitation in contemporary face recognition literature is the reliance on single-split evaluation protocols without statistical uncertainty analysis. Many studies report mean accuracy values without confidence intervals, fold-level variance analysis, or paired statistical testing, limiting the reliability of comparative conclusions. Furthermore, recent research has emphasized that model rankings frequently change under cross-dataset evaluation, indicating that performance superiority on one benchmark may not generalize consistently to different acquisition conditions or demographic distributions (Sharma et al., 2026; Alomar et al., 2025; Palanisamy et al., 2025).

To address these limitations, this study investigates the statistical reliability, supervision sensitivity, and comparative behavior of convolutional, transformer-based, and hybrid face recognition architectures under both Softmax and ArcFace supervision. Specifically, ResNet50, MobileNetV3, DeiT-Small, and a proposed Hybrid multi-branch architecture integrating complementary feature extraction branches are evaluated using five-fold subject-disjoint cross-validation on the Labeled Faces in the Wild (LFW) and FAGEv2 datasets. In addition to identification and verification performance, the study emphasizes fold-level variability, supervision consistency, cross-dataset ranking variability, and loss-function sensitivity.

Unlike prior studies focused primarily on maximizing benchmark accuracy, this work aims to provide a benchmarking framework for evaluating the stability, reproducibility, and supervision sensitivity of face recognition systems under subject-disjoint evaluation conditions. Particular emphasis is placed on fold-level consistency, comparative ranking behavior, and architecture-dependent responses to margin-based supervision. The study further investigates whether angular margin supervision consistently improves discriminative performance across architectures and datasets or whether its effectiveness depends on architectural design and evaluation context.

### 1.1 Research questions

- **RQ1:** How does subject-disjoint cross-validation influence comparative model ranking in face recognition benchmarking?
- **RQ2:** Does ArcFace supervision consistently improve identification and verification performance across architectures and datasets?
- **RQ3:** How stable are model rankings across different evaluation datasets?
- **RQ4:** Can hybrid feature fusion improve discriminative performance relative to individual backbone architectures?

### 1.2 Hypotheses

- **H1:** Comparative model ranking varies across datasets and evaluation settings.
- **H2:** ArcFace supervision does not universally improve performance across all architectures and datasets.
- **H3:** Hybrid feature fusion improves discriminative representation learning relative to individual backbone architectures.
- **H4:** Evaluation outcomes and comparative model rankings vary across datasets.

In summary, although modern face recognition architectures continue to demonstrate strong benchmark performance, insufficient attention has been devoted to fold-level consistency, statistical reliability, ranking variability, and supervision sensitivity. By systematically integrating subject-disjoint cross-validation, fold-level performance analysis, statistical testing, and multi-dataset benchmarking, this study aims to provide a more rigorous and transparent evaluation framework for contemporary face recognition systems.

## 2 Related work

Deep face recognition has advanced significantly through the development of convolutional neural networks, transformer-based architectures, hybrid feature fusion models, and margin-supervised embedding learning. Despite substantial improvements in benchmark accuracy, recent literature increasingly highlights ongoing challenges associated with generalization, reproducibility, statistical reliability, and cross-dataset robustness in practical biometric systems (Haq et al., 2025, 2024).

### 2.1 CNN-based face recognition

CNN-based architectures remain the foundation of most face recognition systems due to their ability to capture local spatial and structural facial features effectively. Residual architectures such as ResNet50 introduced identity skip connections that enabled deeper feature learning while mitigating vanishing gradient problems. These architectures continue to provide strong discriminative performance across identification and verification tasks.

Lightweight convolutional models such as MobileNetV3 were later introduced to reduce computational complexity through depthwise separable convolutions and squeeze-and-excitation attention mechanisms. Such models are particularly relevant for real-time and edge deployment scenarios where computational efficiency is critical. However, lightweight CNNs often face limitations in embedding separability under large intra-class variation or identity-rich datasets, highlighting an ongoing trade-off between efficiency and discriminative capability (Rodrigo et al., 2024; Howard et al., 2021).

### 2.2 Transformer-based face recognition

Transformer-based architectures have recently emerged as powerful alternatives to convolutional models by leveraging self-attention mechanisms to capture long-range dependencies and global contextual information. Vision transformers demonstrate improved robustness to pose variation, occlusion, and contextual variability under several evaluation conditions (Galvn et al., 2025; Rodrigo et al., 2024).

Nevertheless, transformer architectures typically require large-scale training diversity and increased computational resources to achieve stable generalization. Recent studies further indicate that transformer-based systems may exhibit sensitivity to constrained datasets and limited augmentation strategies, particularly in biometric recognition tasks where fine-grained local facial features remain important (Palanisamy et al., 2025; Dosovitskiy et al., 2021).

### 2.3 Hybrid face recognition architectures

To bridge the gap between local feature extraction and global contextual modeling, hybrid architectures combining CNN and transformer components have gained increasing attention. Recent studies demonstrate that hybrid feature fusion can effectively exploit complementary inductive biases, improving robustness and representation learning quality (Alomar et al., 2025; Wu et al., 2022).

Several hybrid approaches integrate convolutional branches for texture modeling with transformer modules for contextual attention, jointly learning local and global facial representations. More recent studies further emphasize computational efficiency by proposing lightweight hybrid designs suitable for deployment on resource-constrained devices while maintaining competitive performance (Alomar et al., 2025; Wu et al., 2022). However, many hybrid architectures introduce increased model complexity, making it challenging to maintain an effective balance between discriminative performance and computational cost.

### 2.4 Margin-based embedding supervision

Margin-based loss functions have become central to modern face recognition systems. Additive angular margin losses such as ArcFace improve embedding separability by enforcing intra-class compactness and inter-class separation within hyperspherical embedding space (Fuad et al., 2021). ArcFace supervision has demonstrated strong verification performance across multiple benchmarks and operating conditions.

However, recent literature suggests that the effectiveness of angular margin supervision may depend on dataset diversity, training conditions, and architectural design. Emerging studies investigating transformer-based and hybrid face recognition systems report that margin-based supervision may exhibit architecture-dependent behavior under constrained or cross-dataset evaluation settings (Zhalgas et al., 2025; Xu et al., 2025). These findings indicate that the relationship between supervision strategy and embedding generalization remains insufficiently understood.

### 2.5 Statistical reliability and cross-dataset evaluation

Recent literature increasingly emphasizes the importance of rigorous and reproducible evaluation protocols in biometric research. Surveys highlight the need for subject-disjoint validation, multi-dataset evaluation, fold-level variance analysis, and statistical significance testing to ensure reliable comparison between face recognition systems (Haq et al., 2025).

Cross-dataset evaluation studies further demonstrate that performance gains observed on one benchmark may not generalize consistently under heterogeneous acquisition conditions, demographic distributions, or preprocessing pipelines (Sharma et al., 2026). Such findings suggest that comparative model ranking is highly sensitive to evaluation context and that single-dataset benchmarking may overestimate generalization capability.

### 2.6 Research gap

Collectively, prior work demonstrates that CNN optimization, transformer-based modeling, hybrid feature fusion, and angular margin supervision each contribute to advances in face recognition performance. However, limited research has systematically examined how supervision strategy, architectural design, and dataset composition jointly influence comparative model behavior, fold-level performance variability, and ranking stability under subject-disjoint evaluation protocols.

Furthermore, many existing studies continue to emphasize peak benchmark performance without sufficient consideration of fold-level consistency, statistical reliability, supervision sensitivity, or ranking variability across datasets. Although angular margin losses such as ArcFace have become widely adopted, their effectiveness across convolutional, transformer-based, and hybrid architectures remains insufficiently understood. Emerging evidence suggests that performance gains may depend on both architectural design and evaluation dataset, highlighting the need for more comprehensive comparative evaluation.

To address these gaps, the present study evaluates CNN, transformer-based, and hybrid face recognition architectures under both Softmax and ArcFace supervision using subject-disjoint cross-validation on the LFW and FAGEv2 datasets. Rather than focusing solely on maximizing benchmark accuracy, the study emphasizes fold-level consistency, evaluation stability, cross-dataset ranking variability, and supervision sensitivity. In addition to identification and verification performance, fold-level statistical analyses are used to assess the consistency of supervision effects across architectures and datasets. The goal is to provide a more transparent assessment of comparative model behavior, robustness, and reproducibility under subject-disjoint evaluation conditions.

## 3 Methodology

### 3.1 Overview

This study investigates the statistical reliability and cross-dataset behavior of convolutional, transformer-based, and hybrid face recognition architectures under controlled evaluation conditions. The experimental framework integrates: (i) standardized preprocessing, (ii) subject-disjoint cross-validation, (iii) multi-branch feature extraction, (iv) embedding projection and normalization, and (v) comparative supervision using both Softmax and ArcFace objectives.

**Figure 1:**
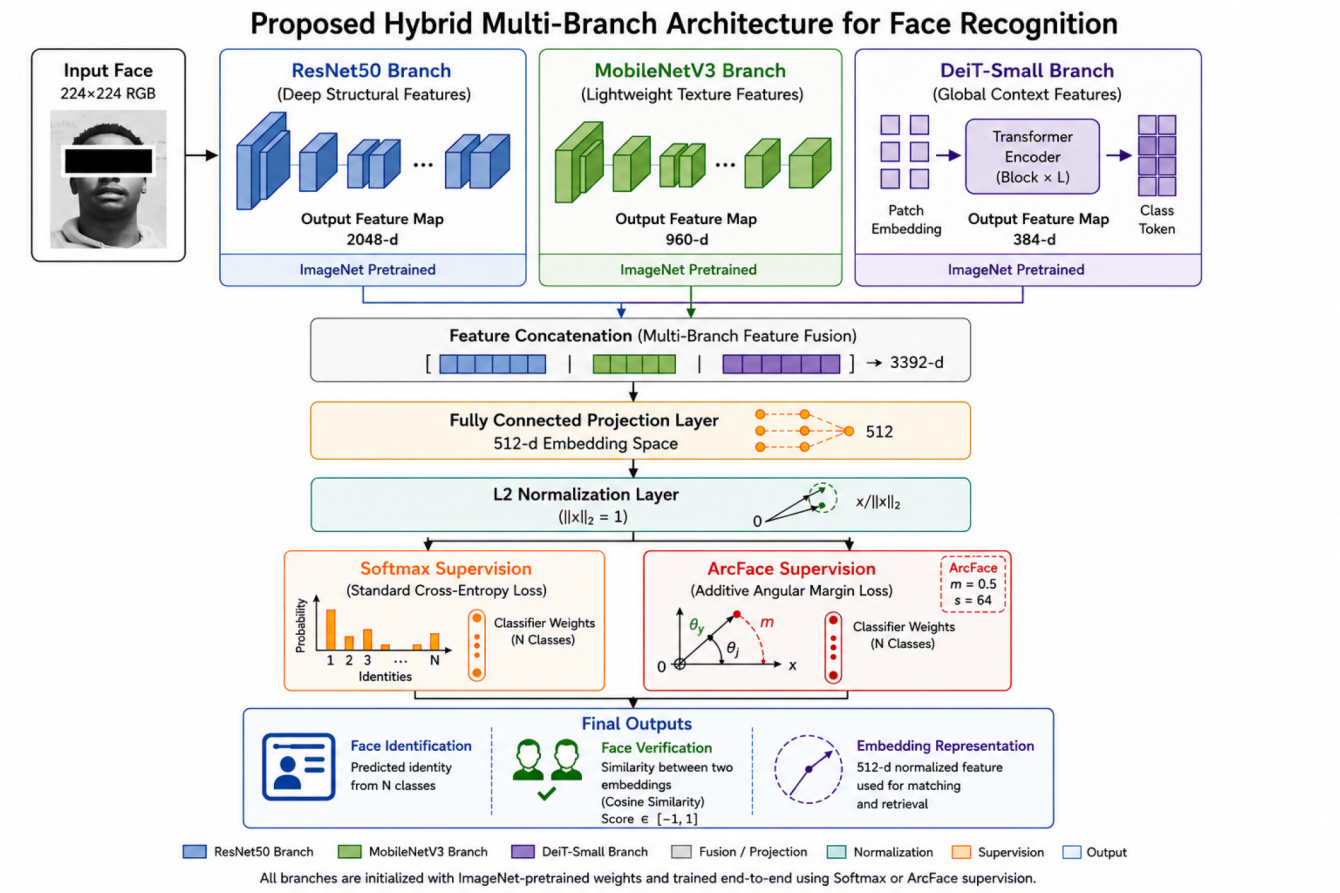
Proposed hybrid multi-branch face recognition framework. ResNet50, MobileNetV3, and DeiT-Small feature representations are fused through feature concatenation and projected into a 512-dimensional normalized embedding space optimized using either Softmax or ArcFace supervision. The learned embeddings are used for face identification and verification.

To ensure fair comparison, all models were evaluated under identical preprocessing, optimization, training, and evaluation conditions. The overall framework is illustrated in Figure 1.

### 3.2 Datasets

The study focuses on controlled statistical evaluation rather than large-scale pre-training performance benchmarking. LFW and FAGEv2 were selected because they provide heterogeneous evaluation conditions while remaining computationally feasible for repeated subject-disjoint cross-validation and fold-level statistical analysis.

#### 3.2.1 LFW dataset

The Labeled Faces in the Wild (LFW) dataset was employed to evaluate unconstrained face recognition performance. LFW contains facial images captured under varying illumination, pose, expression, and background conditions, making it one of the most widely used benchmarks for unconstrained biometric evaluation.

To ensure statistically reliable subject-disjoint validation and sufficient intra-class representation across folds, identities with extremely limited samples were excluded from the experimental subset. Specifically, only identities containing at least 50 facial images were retained for experimentation. Let *n*_*i*_ denote the number of images associated with identity *i*. The filtered identity set was defined as:


$$\begin{array}{l} I_{\text{filtered}}=\left\{i\in I|n_{i}\geq 50\right\} \end{array}$$ (1)

where I represents the complete set of identities in the original LFW dataset. After filtering, the final experimental subset consisted of 217 identities evaluated using five-fold subject-disjoint cross-validation. Although this filtering strategy improves fold stability and ensures sufficient intra-class representation for subject-disjoint evaluation, it modifies the original identity distribution of LFW and should therefore be interpreted as a controlled benchmark subset rather than the complete unconstrained dataset.

#### 3.2.2 FAGEv2 dataset

The FAGEv2 dataset was used to assess cross-dataset generalization and robustness under heterogeneous acquisition conditions. Compared to LFW, FAGEv2 introduces additional challenges associated with constrained identity distributions, reduced intra-class variability, and more difficult cross-identity discrimination. The experimental subset consisted of 219 identities evaluated under the same 5-fold subject-disjoint validation protocol.

### 3.3 Input pre-processing

Each input image is represented as:


$$\begin{array}{l} x\in {\mathbb{R}}^{H\times W\times 3} \end{array}$$ (2)

where *H* and *W* denote spatial dimensions and the three channels correspond to RGB intensity values.

All images were processed using the following standardized pipeline:

- Resize to 224 × 224.
- Random horizontal flipping during training.
- Tensor conversion.
- Normalization using mean and standard deviation values of (0.5, 0.5, 0.5).

No facial landmark alignment or geometric normalization was applied in order to preserve dataset consistency and avoid introducing dataset-specific preprocessing bias.

### 3.4 Subject-disjoint cross-validation

A five-fold subject-disjoint cross-validation protocol was employed to ensure that identities appearing in the training set were completely excluded from the corresponding test fold. This prevents identity leakage and enables statistically reliable estimation of fold-level variance.

For each fold:

- 80% of identities were used for training.
- 20% of identities were used for testing.

Within each training fold, a validation subset was further used for early stopping and model selection.

### 3.5 Baseline architectures

Three baseline architectures representing different deep learning paradigms were evaluated. All architectures were initialized using ImageNet-pre-trained weights prior to fine-tuning. Input preprocessing and normalization were applied consistently across models to ensure comparable feature extraction. For DeiT-Small, the class-token representation was extracted and projected into the shared embedding space before supervision and evaluation.

#### 3.5.1 ResNet50

ResNet50 is a deep residual convolutional architecture employing identity skip connections to facilitate hierarchical feature learning. The final classification layer was removed and replaced with a projection layer generating a 512-dimensional embedding representation.

#### 3.5.2 MobileNetV3-Large

MobileNetV3-Large is a lightweight convolutional architecture utilizing depthwise separable convolutions and squeeze-and-excitation modules for computational efficiency. The final classifier was replaced with a normalized embedding projection layer.

#### 3.5.3 DeiT-Small

DeiT-Small is a transformer-based architecture employing patch embeddings and multi-head self-attention for global contextual representation learning. The class token representation was projected into the shared embedding space.

### 3.6 Proposed hybrid multi-branch architecture

The proposed hybrid architecture integrates complementary feature extraction branches from ResNet50, MobileNetV3, and DeiT-Small to jointly model structural, texture, and contextual facial information.

Let:


$$\begin{array}{ll} f_{r}\left(x\right) & \in {\mathbb{R}}^{d_{r}}\;\text{(ResNet50 features)} \end{array}$$ (3)

$$\begin{array}{ll} f_{m}\left(x\right) & \in {\mathbb{R}}^{d_{m}}\;\text{(MobileNetV3 features)} \end{array}$$ (4)

$$\begin{array}{ll} f_{d}\left(x\right) & \in {\mathbb{R}}^{d_{d}}\;\text{(DeiT-Small features)} \end{array}$$ (5)

where:

- *f*_*r*_(*x*) captures deep structural representations,
- *f*_*m*_(*x*) captures lightweight local texture features,
- *f*_*d*_(*x*) captures global contextual dependencies.

#### 3.6.1 Feature fusion

The fused representation is defined as:


$$\begin{array}{l} f_{h}\left(x\right)=\left[f_{r}\left(x\right)||f_{m}\left(x\right)||f_{d}\left(x\right)\right] \end{array}$$ (6)

where || denotes feature concatenation.

This fusion strategy preserves complementary information from all branches without premature dimensional compression.

### 3.7 Embedding projection and normalization

The fused feature vector is projected into a discriminative embedding space:


$$\begin{array}{l} z=\frac{g\left(f_{h}\left(x\right)\right)}{||g\left(f_{h}\left(x\right)\right){||}_{2}} \end{array}$$ (7)

where:

- *g*(·) denotes the fully connected projection layer,
- *z*∈ℝ^512^ represents the normalized embedding,
- ||·||_2_ denotes the L2 norm.

L2 normalization constrains embeddings to lie on a unit hypersphere, enabling angular-based similarity optimization.

### 3.8 Training objectives

Two supervision strategies were investigated.

#### 3.8.1 Softmax cross-entropy

The baseline classification objective is defined as:


$$\begin{array}{l} L_{\text{softmax}}=-\log\frac{e^{W_{y}^{⊤}z}}{\sum_{j}e^{W_{j}^{⊤}z}} \end{array}$$ (8)

where:

- *W*_*j*_ represents the classifier weight for class *j*,
- *y* denotes the ground-truth identity label.

#### 3.8.2 ArcFace loss

ArcFace introduces an additive angular margin to improve embedding separability.

Let:


$$\begin{array}{l} \cos\left(\theta_{j}\right)=W_{j}^{⊤}z \end{array}$$ (9)

where θ_*j*_ is the angle between embedding *z* and class weight *W*_*j*_.

The target logit becomes:


$$\begin{array}{l} \cos\left(\theta_{y}+m\right) \end{array}$$ (10)

The ArcFace objective is defined as:


$$\begin{array}{l} L_{\text{arc}}=-\log\frac{e^{s\cos\left(\theta_{y}+m\right)}}{e^{s\cos\left(\theta_{y}+m\right)}+\sum_{j\neq y}e^{s\cos\left(\theta_{j}\right)}} \end{array}$$ (11)

where:

- *m* = 0.5 is the angular margin,
- *s* = 64 is the scaling factor.

This formulation increases inter-class separation while reducing intra-class embedding variance.

### 3.9 Training configuration

All models were trained under identical optimization conditions to ensure comparability.

- Optimizer: AdamW.
- Learning rate: 3 × 10^−4^.
- Batch size: 32.
- Maximum epochs: 100.
- Early stopping patience: 5 epochs.

Training was terminated if validation Top-1 accuracy failed to improve for five consecutive epochs. The best-performing checkpoint was retained for evaluation. No explicit learning rate scheduler was employed during training.

### 3.10 Evaluation metrics

#### 3.10.1 Identification performance

Top-1 identification accuracy was computed as:


$$\begin{array}{l} \text{Top-1}=\frac{1}{N}\overset{N}{\underset{i=1}{\sum }}1\left({ŷ}_{i}=y_{i}\right) \end{array}$$ (12)

where **1**(·) denotes the indicator function.

#### 3.10.2 Verification performance

Verification performance was evaluated using pairwise comparisons between normalized face embeddings. Within each subject-disjoint test fold, all unique image pairs were generated. Pairs belonging to the same identity were treated as genuine pairs, whereas pairs belonging to different identities were treated as impostor pairs.

For each pair, cosine similarity was computed as:


$$\begin{array}{l} s_{ij}=z_{i}^{⊤}z_{j} \end{array}$$ (13)

where *z*_*i*_ and *z*_*j*_ denote the L2-normalized embeddings of two facial images. Higher similarity scores indicate greater similarity between the corresponding facial representations and therefore stronger evidence that both images belong to the same identity.

The resulting genuine and impostor similarity scores were used to generate Receiver Operating Characteristic (ROC) curves by varying the decision threshold. From these score distributions, verification performance was quantified using the Area Under the Curve (AUC) and Equal Error Rate (EER).

##### 3.10.2.1 True positive rate

The True Positive Rate (TPR), also referred to as the genuine acceptance rate, was computed as:


$$\begin{array}{l} \text{TPR}\left(\tau \right)=\frac{\text{TP}}{\text{TP}+\text{FN}} \end{array}$$ (14)

where TP and FN denote the numbers of correctly accepted and incorrectly rejected genuine pairs, respectively.

##### 3.10.2.2 False positive rate

The False Positive Rate (FPR), also referred to as the false acceptance rate, was computed as:


$$\begin{array}{l} \text{FPR}\left(\tau \right)=\frac{\text{FP}}{\text{FP}+\text{TN}} \end{array}$$ (15)

where FP and TN denote the numbers of incorrectly accepted impostor pairs and correctly rejected impostor pairs, respectively.

##### 3.10.2.3 Equal error rate

Equal Error Rate (EER) was computed at the operating threshold τ^*^ where the false acceptance and false rejection rates are equal:


$$\begin{array}{l} \text{FPR}\left(\tau^{*}\right)=1-\text{TPR}\left(\tau^{*}\right) \end{array}$$ (16)

Lower EER values indicate improved verification performance and greater separation between genuine and impostor similarity distributions.

#### 3.10.3 Computational complexity

Computational efficiency was assessed using:

- GFLOPs,
- Parameter count.

### 3.11 Statistical analysis

Fold-level performance statistics were aggregated across five subject-disjoint folds to evaluate model stability and reduce the risk of identity leakage between training and testing partitions. A five-fold cross-validation protocol was selected to balance computational feasibility with the need to assess fold-level variability and comparative consistency across architectures and supervision strategies. Similar fold configurations are widely adopted in biometric benchmarking studies to provide reproducible performance estimation under practical training constraints.

For each model and supervision strategy, the mean and standard deviation of Top-1 identification accuracy were computed across folds to quantify performance variability and comparative stability. To further assess supervision sensitivity, a fold consistency analysis was performed by recording the number of folds in which ArcFace achieved higher identification accuracy than Softmax for each architecture.

Given the limited number of cross-validation folds (*n* = 5), Wilcoxon signed-rank tests were employed to provide a non-parametric assessment of fold-level performance differences between Softmax and ArcFace supervision. The Wilcoxon test was selected because it does not require normality assumptions and is more appropriate for small paired samples than conventional parametric alternatives.

The statistical analysis was intended to complement the primary benchmarking results by providing additional insight into performance stability, supervision consistency, and comparative model behavior under subject-disjoint evaluation conditions. Given the limited number of folds, the statistical analysis should be interpreted primarily as an indication of comparative consistency rather than definitive inferential evidence. Future work may investigate larger-scale evaluation protocols and alternative statistical procedures to strengthen inferential conclusions.

### 3.12 Methodological summary

The proposed framework integrates controlled preprocessing, subject-disjoint cross-validation, hybrid feature fusion, dual supervision strategies, and fold-level statistical evaluation within a unified benchmarking pipeline. By combining identification and verification assessment, multi-dataset benchmarking, fold-level consistency analysis, and supervision sensitivity evaluation, the methodology provides a reproducible framework for examining comparative model behavior, ranking variability, and performance stability under subject-disjoint evaluation conditions.

Rather than focusing solely on peak benchmark accuracy, the framework emphasizes evaluation reliability, supervision consistency, and comparative ranking behavior across architectures and datasets. This enables a more transparent assessment of how architectural design and supervision strategy influence face recognition performance under controlled benchmarking conditions.

## 4 Results

### 4.1 Overview of experimental findings

This section presents the identification, verification, computational complexity, and fold-level statistical evaluation results obtained using five-fold subject-disjoint cross-validation on the LFW and FAGEv2 datasets. Comparative analysis was conducted across ResNet50, MobileNetV3, DeiT-Small, and the proposed Hybrid multi-branch architecture under both Softmax and ArcFace supervision.

The experiments evaluate not only absolute performance but also supervision sensitivity, fold-level consistency, ranking variability, and computational trade-offs. Identification performance is assessed using Top-1 accuracy, while verification performance is evaluated using Area Under the ROC Curve (AUC) and Equal Error Rate (EER). Additional fold-level analyses were performed to examine performance stability and the consistency of supervision effects across architectures.

### 4.2 Performance on LFW

Table 1 summarizes the five-fold subject-disjoint cross-validation results on the LFW dataset. Under Softmax supervision, the Hybrid architecture achieved the highest Top-1 identification accuracy (62.4%), followed by DeiT-Small (61.6%) and MobileNetV3 (59.5%). Under ArcFace supervision, MobileNetV3 achieved the highest identification accuracy (62.1%), while ResNet50 achieved the largest improvement relative to its Softmax counterpart.

**Table 1:** Five-fold subject-disjoint cross-validation performance on the LFW dataset.

Model	Top-1 (%)	AUC	EER
ResNet50-Softmax	43.4	0.788	0.288
ResNet50-ArcFace	50.8	0.816	0.261
MobileNetV3-Softmax	59.5	0.877	0.204
MobileNetV3-ArcFace	62.1	0.865	0.214
DeiT-Small-Softmax	61.6	**0.905**	**0.159**
DeiT-Small-ArcFace	58.4	0.857	0.225
Hybrid-Softmax	**62.4**	0.892	0.183
Hybrid-ArcFace	61.5	0.874	0.209

Bold values indicate the best-performing result for each evaluation metric (highest Top-1 accuracy and AUC, and lowest Equal Error Rate (EER)).

Verification performance exhibited a partially different trend. DeiT-Small-Softmax achieved the highest verification performance, obtaining an AUC of 0.905 and the lowest EER of 0.159. Hybrid-Softmax achieved the second-highest verification performance, with an AUC of 0.892 and EER of 0.183.

**Figure 2:**
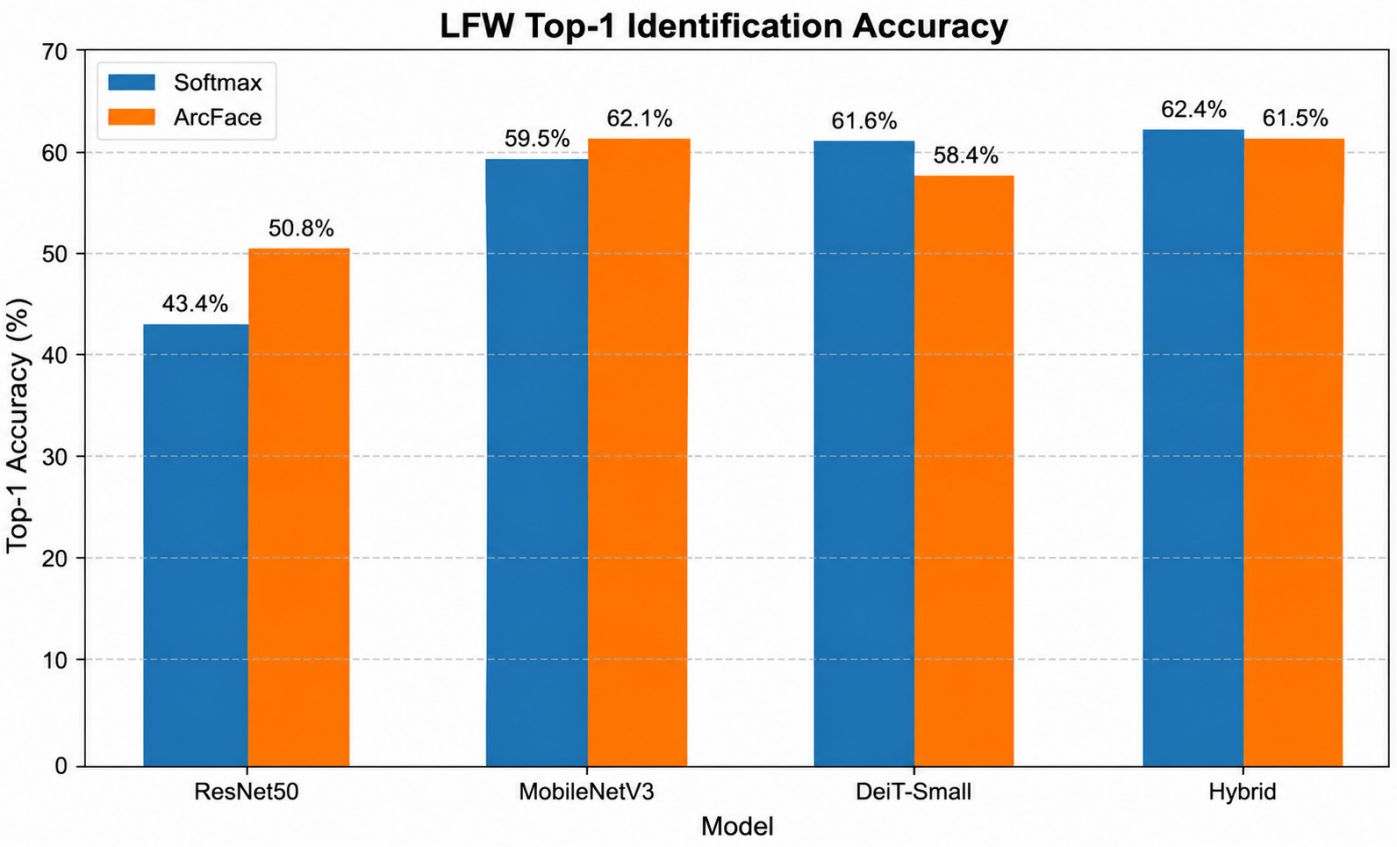
Top-1 identification accuracy on the LFW dataset.

**Figure 3:**
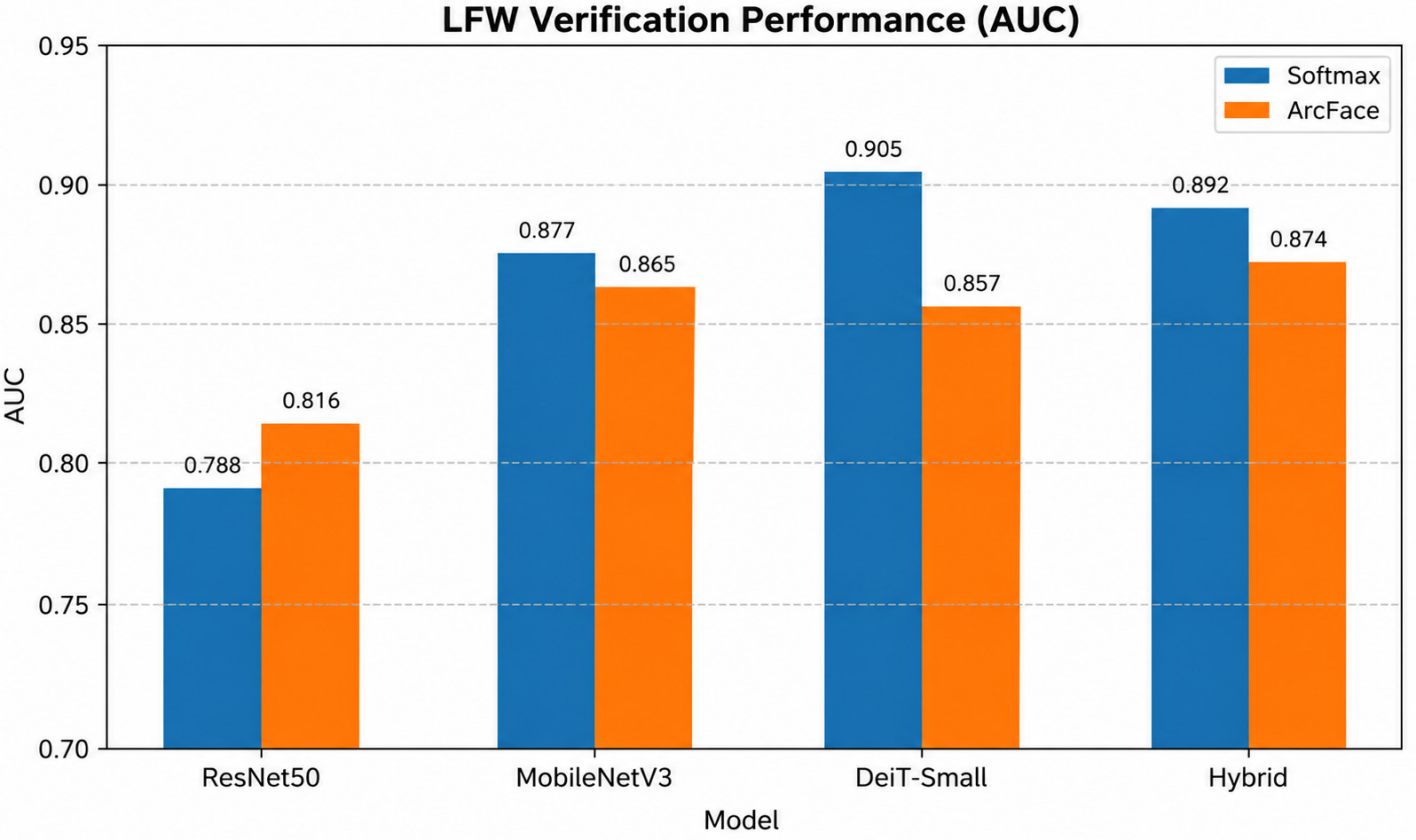
Verification AUC on the LFW dataset.

**Figure 4:**
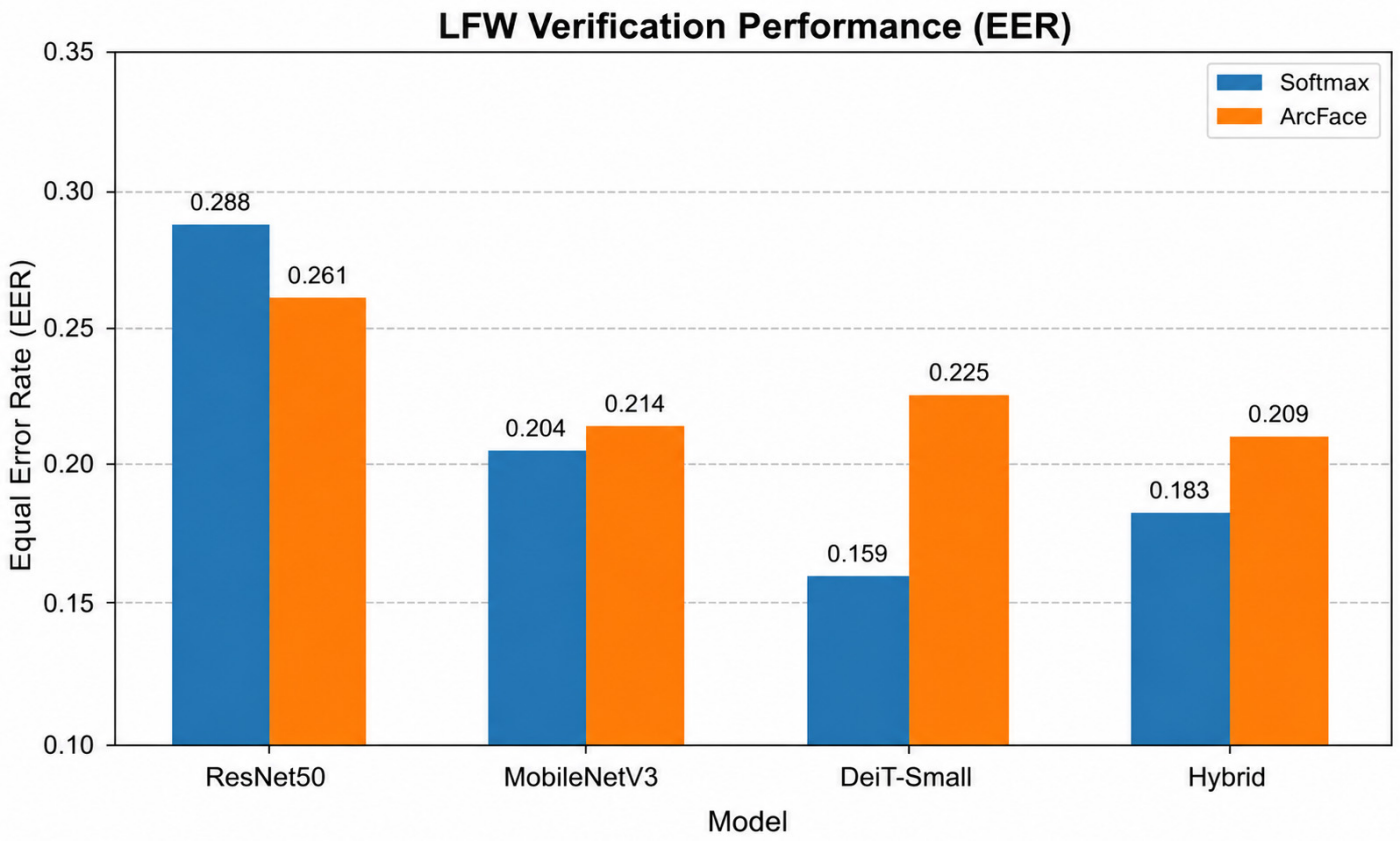
Equal Error Rate (EER) on the LFW dataset.

Figures 2–4 provide a visual comparison of identification and verification performance on the LFW dataset.

### 4.3 Fold-level supervision sensitivity analysis on LFW

To further examine fold-level performance behavior, Top-1 identification accuracy was analyzed across the five subject-disjoint cross-validation folds. Table 2 summarizes the mean identification accuracy, standard deviation, fold consistency score, and Wilcoxon signed-rank test results for each architecture.

**Table 2:** Fold-level supervision sensitivity analysis on the LFW dataset.

Model	Softmax mean ± SD	ArcFace mean ± SD	ArcFace better folds	Wilcoxon *p*
ResNet50	0.480 ± 0.055	0.515 ± 0.042	5/5	0.0625
MobileNetV3	0.622 ± 0.042	0.626 ± 0.045	3/5	0.6250
DeiT-Small	0.634 ± 0.037	0.554 ± 0.043	0/5	0.0625
Hybrid	0.654 ± 0.040	0.634 ± 0.047	0/5	0.0625

ResNet50 achieved higher Top-1 identification accuracy under ArcFace supervision in all five folds. MobileNetV3 achieved higher accuracy under ArcFace supervision in three of the five folds. DeiT-Small and the Hybrid architecture achieved higher identification accuracy under Softmax supervision in all five folds.

The Wilcoxon signed-rank tests did not reach the conventional significance threshold of *p* < 0.05 for any architecture. However, the fold consistency scores indicate stable fold-level performance differences between supervision strategies.

**Figure 5:**
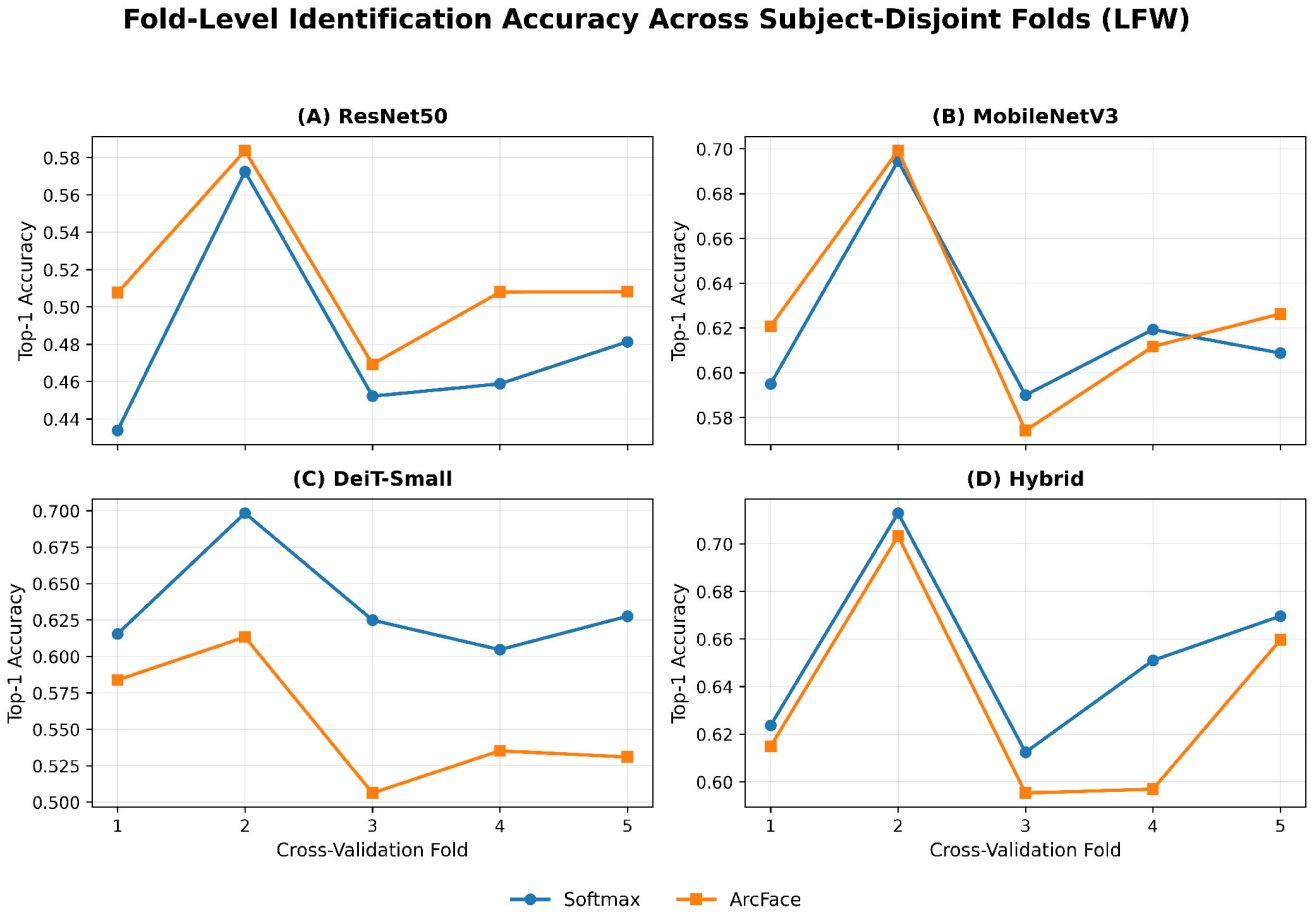
Fold-level Top-1 identification accuracy across the five subject-disjoint cross-validation folds on the LFW dataset. Softmax and ArcFace supervision are compared for **(A)** ResNet50, **(B)** MobileNetV3, **(C)** DeiT-Small, and **(D)** the proposed Hybrid architecture.

Figure 5 presents fold-level Top-1 identification accuracy across the five subject-disjoint cross-validation folds. The figure illustrates the fold-to-fold variation observed under Softmax and ArcFace supervision for each architecture.

### 4.4 Performance on FAGEv2

Table 3 presents the five-fold subject-disjoint cross-validation results on the FAGEv2 dataset. Compared with LFW, lower identification and verification performance was observed across all architectures and supervision strategies on the FAGEv2 dataset.

**Table 3:** Five-fold subject-disjoint cross-validation performance on the FAGEv2 dataset.

Model	Top-1 (%)	AUC	EER
ResNet50-Softmax	19.6	0.663	0.387
ResNet50-ArcFace	21.8	0.678	0.381
MobileNetV3-Softmax	35.5	0.742	0.321
MobileNetV3-ArcFace	30.0	0.732	0.334
DeiT-Small-Softmax	38.0	0.816	0.261
DeiT-Small-ArcFace	36.5	0.760	0.310
Hybrid-Softmax	**38.0**	**0.825**	**0.251**
Hybrid-ArcFace	35.1	0.767	0.307

Bold values indicate the best-performing result for each evaluation metric (highest Top-1 accuracy and AUC, and lowest Equal Error Rate (EER)).

Under Softmax supervision, Hybrid-Softmax and DeiT-Small-Softmax jointly achieved the highest Top-1 identification accuracy (38.0%), followed by MobileNetV3-Softmax (35.5%). Under ArcFace supervision, DeiT-Small achieved the highest Top-1 identification accuracy (36.5%), followed by the Hybrid architecture (35.1%).

Verification performance followed a similar trend. Hybrid-Softmax achieved the highest AUC (0.825) and the lowest EER (0.251), while DeiT-Small-Softmax achieved the second-highest verification performance with an AUC of 0.816 and EER of 0.261.

**Figure 6:**
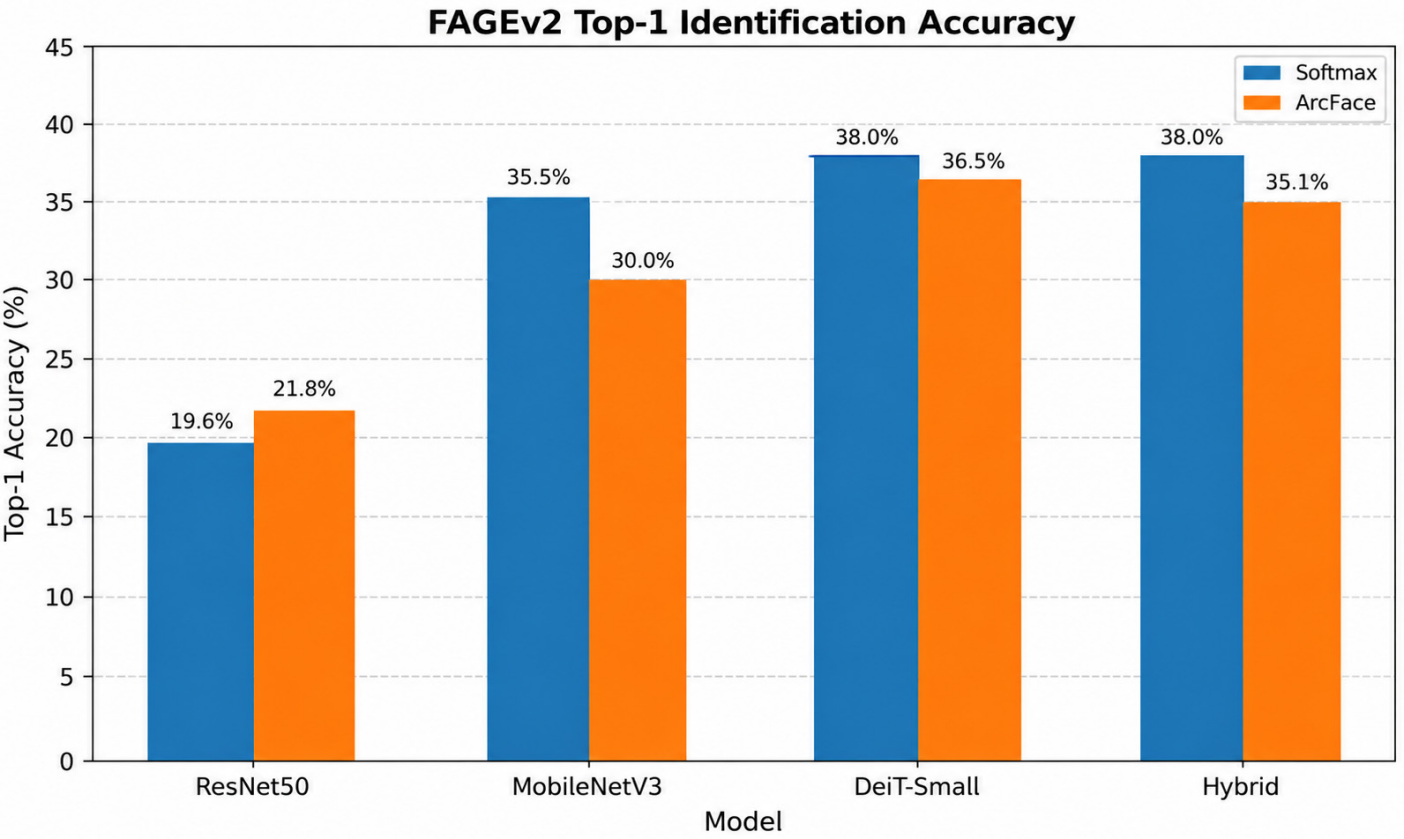
Top-1 identification accuracy on the FAGEv2 dataset.

**Figure 7:**
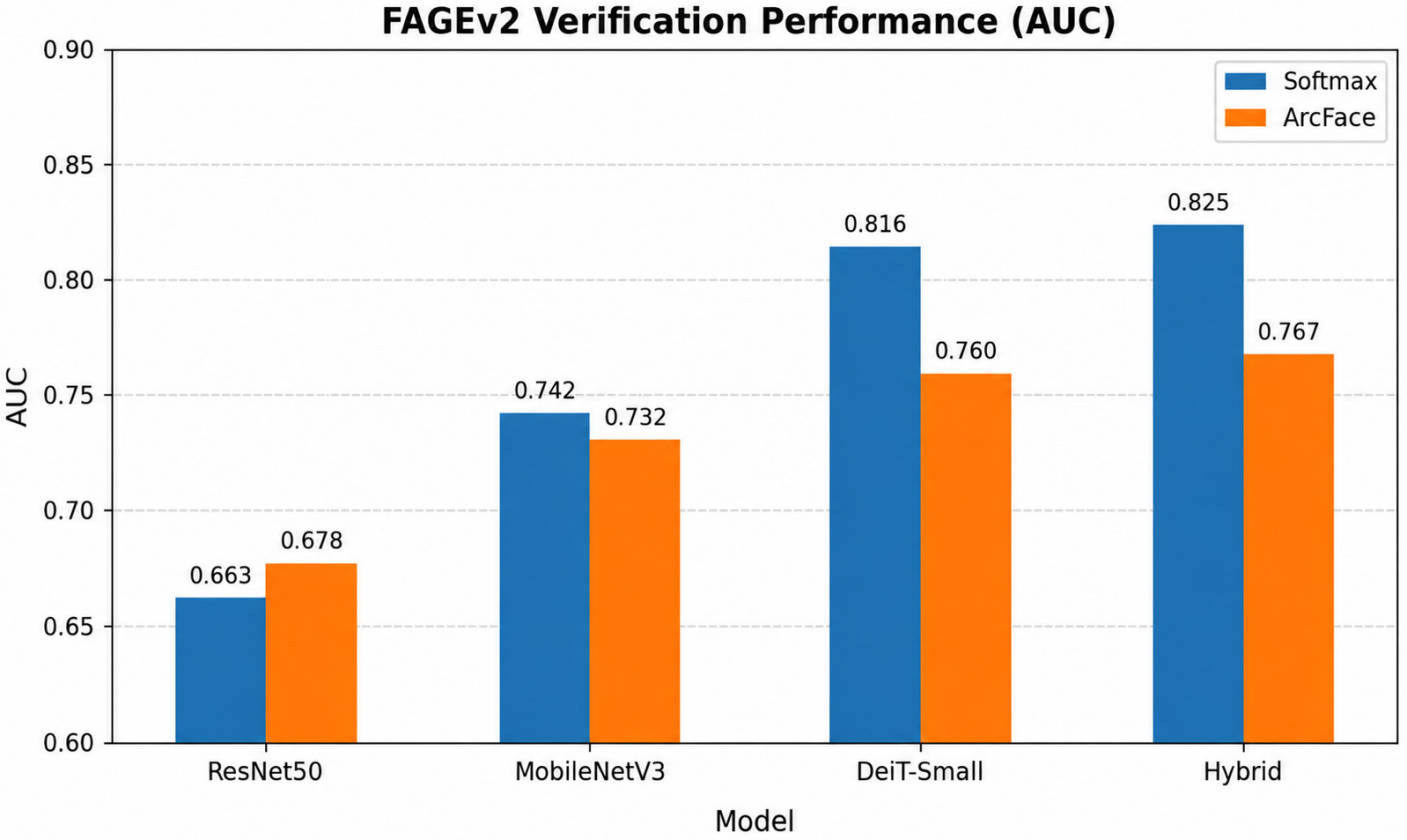
Verification AUC on the FAGEv2 dataset.

**Figure 8:**
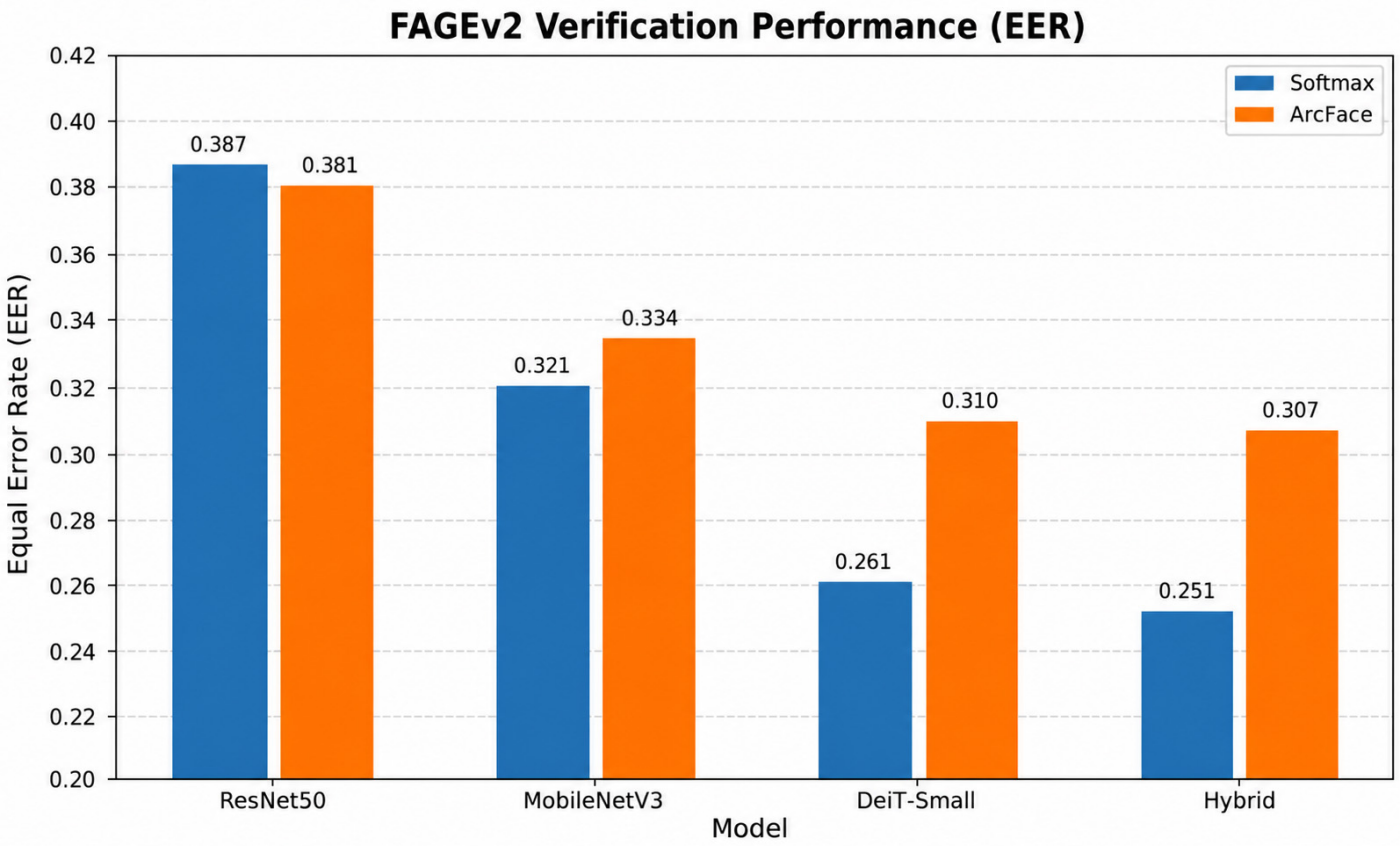
Equal Error Rate (EER) on the FAGEv2 dataset.

Figures 6–8 provide a visual comparison of identification and verification performance on the FAGEv2 dataset.

### 4.5 Fold-level supervision sensitivity analysis on FAGEv2

To further examine fold-level performance behavior on the FAGEv2 dataset, Top-1 identification accuracy was analyzed across the five subject-disjoint cross-validation folds. Table 4 summarizes the mean identification accuracy, standard deviation, fold consistency score, and Wilcoxon signed-rank test results for each architecture.

**Table 4:** Fold-level supervision sensitivity analysis on the FAGEv2 dataset.

Model	Softmax mean ± SD	ArcFace mean ± SD	ArcFace better folds	Wilcoxon *p*
ResNet50	0.182 ± 0.018	0.196 ± 0.024	4/5	0.1250
MobileNetV3	0.314 ± 0.025	0.281 ± 0.016	0/5	0.0625
DeiT-Small	0.314 ± 0.045	0.316 ± 0.038	3/5	0.8125
Hybrid	0.331 ± 0.044	0.326 ± 0.027	2/5	0.8125

ResNet50 achieved higher Top-1 identification accuracy under ArcFace supervision in four of the five folds. DeiT-Small achieved higher accuracy under ArcFace supervision in three folds, while the Hybrid architecture achieved higher accuracy under ArcFace supervision in two folds. MobileNetV3 achieved higher identification accuracy under Softmax supervision in all five folds.

The Wilcoxon signed-rank tests did not reach the conventional significance threshold of *p* < 0.05 for any architecture.

**Figure 9:**
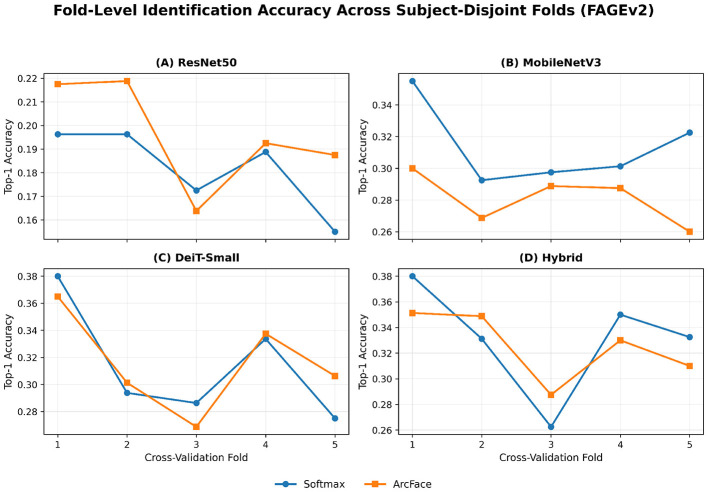
Fold-level Top-1 identification accuracy across the five subject-disjoint cross-validation folds on the FAGEv2 dataset. Softmax and ArcFace supervision are compared for **(A)** ResNet50, **(B)** MobileNetV3, **(C)** DeiT-Small, and **(D)** the proposed Hybrid architecture.

Figure 9 presents fold-level Top-1 identification accuracy across the five subject-disjoint cross-validation folds. The figure illustrates the fold-to-fold variation observed under Softmax and ArcFace supervision for each architecture.

### 4.6 Computational complexity analysis

Table 5 summarizes the computational complexity of the evaluated architectures in terms of floating-point operations (GFLOPs) and parameter count. MobileNetV3 exhibited the lowest computational complexity, requiring only 0.22 GFLOPs and 4.18 million parameters, whereas the Hybrid architecture exhibited the highest complexity, requiring 8.59 GFLOPs and 30.06 million parameters.

**Table 5:** Model complexity comparison.

Model	GFLOPs	Parameters (M)
ResNet50	4.11	25.56
MobileNetV3	0.22	4.18
DeiT-Small	4.63	21.96
Hybrid	8.59	30.06

**Figure 10:**
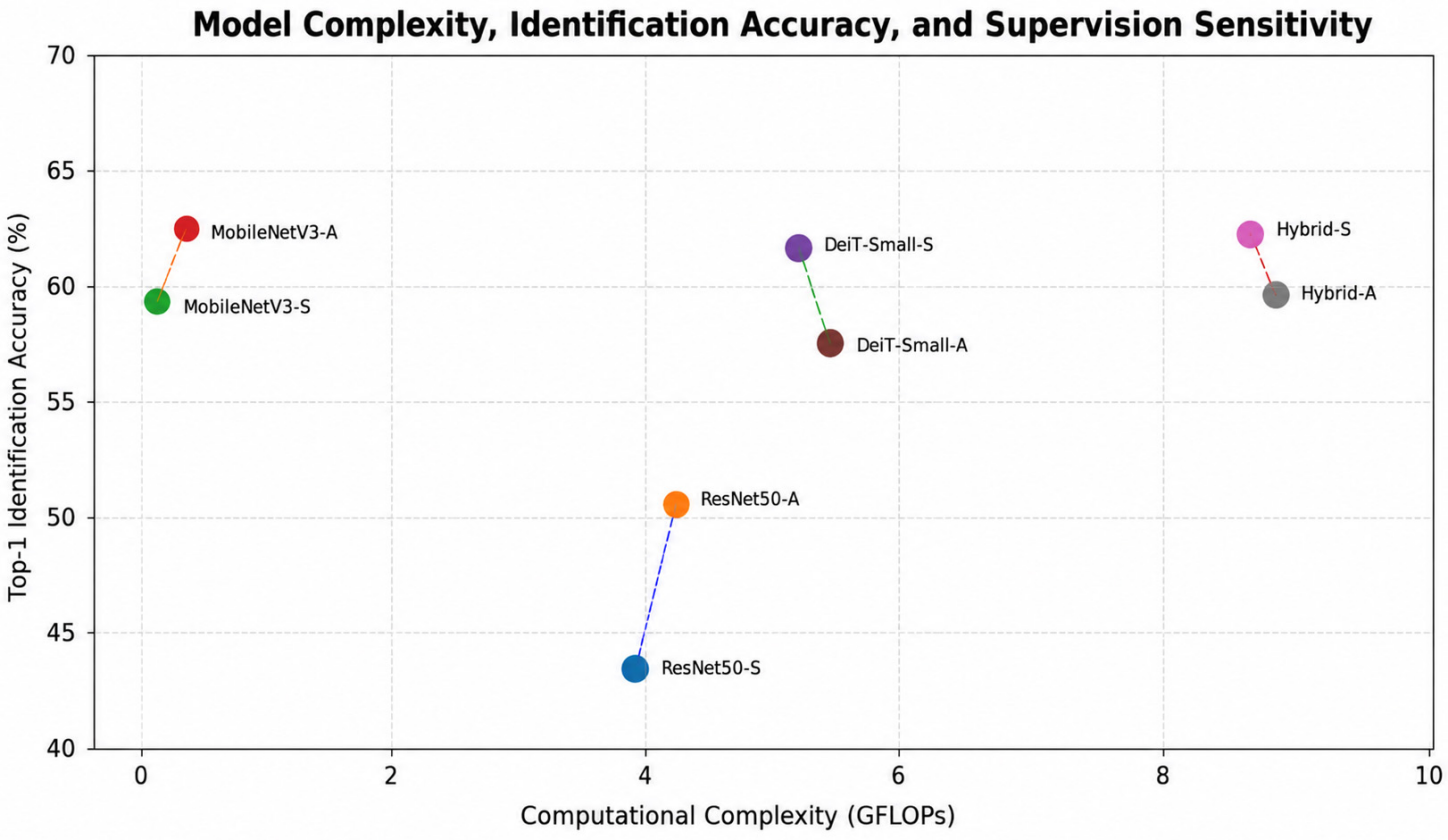
Relationship between computational complexity, identification accuracy, and supervision strategy on the LFW dataset. Bubble size is proportional to parameter count. Softmax (S) and ArcFace (A) results are shown separately for each architecture, while dashed lines indicate the performance difference between supervision strategies.

Figure 10 presents the relationship between computational complexity, Top-1 identification accuracy, and supervision strategy on the LFW dataset. Bubble size is proportional to parameter count, while the paired Softmax and ArcFace points illustrate supervision sensitivity for each architecture.

**Figure 11:**
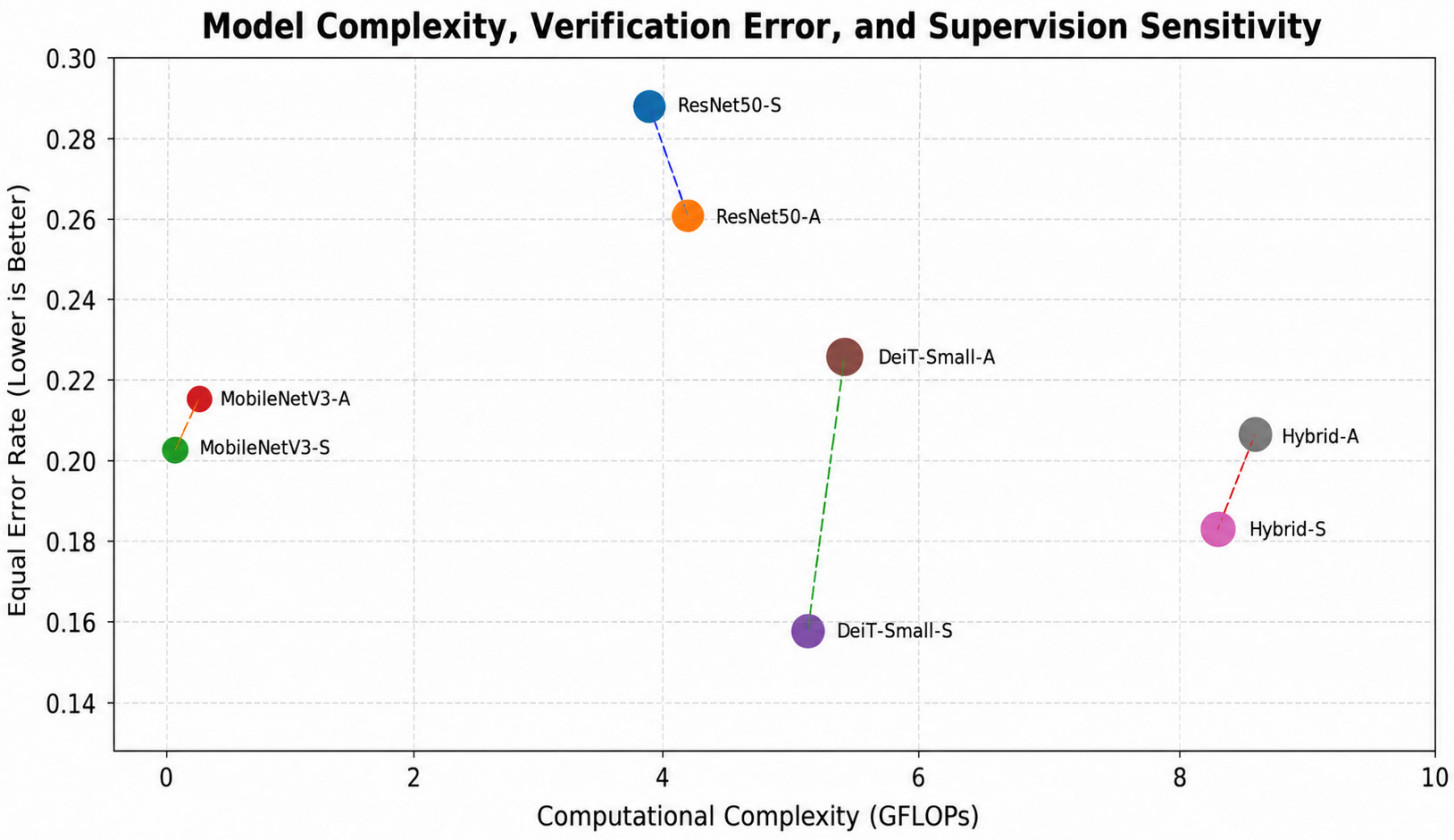
Relationship between computational complexity, verification error, and supervision strategy on the LFW dataset. Bubble size is proportional to parameter count. Softmax (S) and ArcFace (A) results are shown separately for each architecture, while dashed lines indicate the performance difference between supervision strategies.

Figure 11 presents the relationship between computational complexity, verification error, and supervision strategy. Bubble size is proportional to parameter count, while the paired Softmax and ArcFace points illustrate supervision sensitivity for each architecture.

Overall, the results demonstrate substantial variation in identification performance, verification performance, fold-level supervision behavior, and computational complexity across architectures and datasets. Differences were observed between Softmax and ArcFace supervision under both LFW and FAGEv2 evaluation, while fold-level analyses revealed architecture-dependent supervision sensitivity. The computational complexity analysis further highlighted the trade-offs between recognition performance, model size, and computational cost. These findings provide the empirical basis for the subsequent discussion of supervision sensitivity, cross-dataset behavior, and architecture-specific performance characteristics.

## 5 Discussion

This study investigated the statistical reliability, cross-dataset behavior, and supervision sensitivity of convolutional, transformer-based, and hybrid face recognition architectures under subject-disjoint evaluation. The findings provide important insight into how architectural design, supervision strategy, and dataset characteristics jointly influence discriminative performance and benchmarking reliability.

### 5.1 Performance behavior across architectures

The experimental results demonstrate that no single architecture consistently achieved the highest performance across all datasets and evaluation settings. On the LFW dataset, Hybrid-Softmax achieved the highest Top-1 identification accuracy (62.4%), while DeiT-Small-Softmax achieved the strongest verification performance with the highest AUC (0.905) and lowest EER (0.159) (Table 1; Figures 2–4). On the FAGEv2 dataset, Hybrid-Softmax and DeiT-Small-Softmax jointly achieved the highest identification accuracy (38.0%), while Hybrid-Softmax achieved the strongest verification performance (Table 3; Figures 6–8).

The observed changes in relative performance across datasets indicate that architecture behavior is influenced by evaluation context. Models that achieved strong performance on one dataset did not necessarily maintain the same relative ranking on another dataset. These observations suggest that comparative benchmarking outcomes depend not only on architectural design but also on dataset characteristics and evaluation conditions.

The findings support Hypothesis H1 and are consistent with recent studies emphasizing the importance of evaluating face recognition systems across multiple datasets to obtain a more comprehensive assessment of model behavior and robustness.

### 5.2 Effectiveness of ArcFace supervision

A central finding of this study is that ArcFace supervision did not consistently improve performance across architectures and datasets. The fold-level analyses revealed that the effect of ArcFace varied substantially depending on both architectural design and evaluation dataset (Tables 2, 4; Figures 5, 9).

On the LFW dataset, ArcFace consistently improved ResNet50 performance across all evaluation folds and provided modest benefits for MobileNetV3. In contrast, DeiT-Small and the Hybrid architecture achieved higher identification accuracy under Softmax supervision in every fold (Table 2; Figure 5). A different pattern emerged on the FAGEv2 dataset, where ArcFace improved ResNet50 performance in four of the five folds and DeiT-Small performance in three folds, while MobileNetV3 consistently achieved higher performance under Softmax supervision (Table 4; Figure 9).

These findings challenge the common assumption that additive angular margin supervision universally outperforms conventional Softmax optimization. Instead, the results suggest that the effectiveness of margin-based embedding learning depends on the interaction between architecture type, dataset characteristics, and representation learning behavior.

One possible explanation is that angular margin constraints influence feature distributions differently across convolutional, transformer-based, and hybrid architectures. While margin-based supervision may enhance class separation for certain representations, it may also impose constraints that are not equally beneficial across all feature extraction paradigms. The differing behavior observed between LFW and FAGEv2 further indicates that dataset characteristics play an important role in determining the effectiveness of supervision strategies.

Overall, the findings support Hypothesis H2 and demonstrate that supervision strategy should be evaluated within the context of both architecture design and dataset characteristics rather than assumed to provide universal performance improvements.

### 5.3 Hybrid feature fusion and representation learning

The proposed Hybrid architecture demonstrated competitive performance across both datasets and supervision strategies. On the LFW dataset, Hybrid-Softmax achieved the highest Top-1 identification accuracy among all evaluated models while maintaining strong verification performance (Table 1; Figures 2–4). On the FAGEv2 dataset, Hybrid-Softmax jointly achieved the highest identification accuracy and obtained the strongest verification performance in terms of both AUC and EER (Table 3; Figures 6–8).

These results suggest that combining complementary feature extraction mechanisms can provide robust discriminative representations across different evaluation settings. By integrating convolutional and transformer-based branches, the Hybrid architecture leverages local feature extraction, hierarchical representation learning, and global contextual modeling within a unified framework.

The performance of the Hybrid model was particularly notable under Softmax supervision, where it consistently ranked among the strongest-performing architectures on both datasets. However, the fold-level analyses revealed that Hybrid performance was sensitive to the choice of supervision strategy, with ArcFace failing to provide consistent improvements across either dataset (Tables 2, 4).

Overall, the findings support Hypothesis H3 and are consistent with recent studies suggesting that hybrid representation learning can benefit face recognition by combining complementary inductive biases from multiple architectural paradigms. At the same time, the results highlight the importance of considering both architectural design and supervision strategy when developing high-performance biometric systems.

### 5.4 Cross-dataset performance variability

A notable outcome of this study was the variation in model behavior across datasets. Although the same architectures, training procedures, and supervision strategies were applied, substantial differences were observed between the LFW and FAGEv2 evaluations (Tables 1, 3).

Across all architectures, identification and verification performance were lower on FAGEv2 than on LFW. In addition to the reduction in absolute performance, changes were observed in the relative ranking of architectures across datasets (Tables 1, 3). For example, MobileNetV3 achieved the highest identification accuracy under ArcFace supervision on LFW, whereas DeiT-Small achieved the highest identification accuracy under ArcFace supervision on FAGEv2. Similarly, the relative ordering of architectures under Softmax supervision differed between datasets.

The fold-level supervision analyses further revealed that the effect of ArcFace varied across datasets (Tables 2, 4). While ArcFace consistently improved ResNet50 performance on both datasets, different supervision patterns were observed for MobileNetV3, DeiT-Small, and the Hybrid architecture.

Collectively, the results demonstrate that conclusions regarding model superiority can vary depending on the evaluation dataset. These observations support Hypothesis H4 and reinforce the importance of multi-dataset evaluation when assessing face recognition systems.

The findings are consistent with recent recommendations in biometric research advocating the use of heterogeneous benchmark datasets and subject-disjoint evaluation protocols to obtain a more reliable assessment of model robustness and generalization behavior.

### 5.5 Computational complexity and efficiency trade-offs

The computational analysis revealed important trade-offs between model complexity and recognition performance. As shown in Table 5, MobileNetV3 required the fewest parameters (4.18M) and lowest computational cost (0.22 GFLOPs), whereas the proposed Hybrid architecture exhibited the highest computational complexity, requiring 30.06M parameters and 8.59 GFLOPs.

Despite its substantially lower computational requirements, MobileNetV3 achieved competitive identification and verification performance across both datasets (Tables 1, 3). This finding highlights the effectiveness of lightweight architectures for resource-constrained deployment environments where computational efficiency is a primary consideration.

In contrast, the Hybrid architecture consistently achieved among the strongest identification and verification results across both datasets (Tables 1, 3). However, these performance gains were accompanied by increased computational requirements, reflecting the additional complexity introduced by multi-branch feature extraction and feature fusion.

The complexity-performance relationships illustrated in Figures 10, 11, together with the complexity measurements reported in Table 5, further demonstrate that recognition performance is influenced by more than computational complexity alone. The bubble visualizations simultaneously highlight computational cost, parameter count, and supervision sensitivity. Although DeiT-Small and ResNet50 exhibited comparable computational requirements (Table 5), notable differences were observed in both identification and verification performance (Tables 1, 3). Similarly, the supervision-specific performance trajectories shown in Figures 10, 11 indicate that changes in optimisation strategy can influence recognition outcomes without altering model complexity.

These observations suggest that architectural design, representation learning strategy, and supervision choice collectively influence recognition performance beyond what can be explained by model size alone. As illustrated in Figures 10, 11, architectures with similar computational requirements may exhibit substantially different identification and verification performance. Consequently, selecting an appropriate face recognition architecture requires balancing recognition performance, computational cost, parameter efficiency, and deployment requirements rather than optimizing a single performance metric.

### 5.6 Statistical reliability and benchmarking implications

A major contribution of this study lies in demonstrating the value of fold-level evaluation and supervision consistency analysis in face recognition benchmarking. While average performance metrics provide a useful summary of model behavior, the fold-level analyses revealed important differences in supervision sensitivity and performance stability that would not be apparent from mean performance alone.

The fold consistency analyses (Tables 2, 4) showed that several supervision effects were highly consistent across evaluation folds. For example, ArcFace improved ResNet50 performance across all folds on LFW and four of five folds on FAGEv2, whereas Softmax consistently outperformed ArcFace for the Hybrid architecture on LFW and for MobileNetV3 on FAGEv2. These patterns remained evident despite the Wilcoxon signed-rank tests not reaching conventional significance thresholds.

The findings demonstrate that subject-disjoint cross-validation, fold-level variance analysis, Wilcoxon signed-rank testing, and fold consistency assessment provide a more informative evaluation framework than single-split benchmarking alone. Such approaches help reveal performance stability, supervision sensitivity, and ranking variability that may otherwise remain hidden when only average benchmark scores are reported.

Consequently, future face recognition studies should complement traditional performance metrics with fold-level statistical analyses and multi-dataset evaluation protocols to improve benchmarking transparency and reproducibility.

### 5.7 Practical implications for biometric systems

The findings of this study have several implications for the deployment of face recognition systems in practical biometric applications. The results demonstrate that architecture selection should consider not only peak identification accuracy but also verification performance, computational efficiency, supervision sensitivity, and performance consistency across evaluation settings.

For resource-constrained environments, MobileNetV3 provides an attractive balance between computational efficiency and recognition performance. As shown in Table 5, MobileNetV3 achieved competitive identification and verification performance while requiring substantially fewer parameters and computational operations than the other evaluated architectures.

For applications where recognition accuracy is prioritized over computational efficiency, the Hybrid architecture offers a strong alternative. The model consistently achieved among the strongest identification and verification results across both datasets (Tables 1, 3), demonstrating the potential benefits of combining complementary feature extraction paradigms.

The observed differences between Softmax and ArcFace supervision further indicate that optimization objectives should be selected based on both architectural design and deployment conditions. The fold-level analyses (Tables 2, 4) showed that supervision effects varied considerably across architectures and datasets, suggesting that a supervision strategy that performs well in one setting may not necessarily provide similar benefits in another.

Although fairness and demographic bias were not explicitly evaluated in this study, the observed variability across datasets highlights the importance of assessing model behavior across diverse populations and operational conditions. Future deployment-oriented studies should therefore incorporate fairness-aware evaluation protocols and demographic performance analysis to ensure reliable and equitable biometric system performance.

### 5.8 Discussion summary

Overall, the findings demonstrate that architecture design, supervision strategy, and dataset characteristics jointly influence face recognition performance. The experimental results showed that no single architecture consistently dominated across all evaluation settings, while substantial differences were observed in supervision behavior and model ranking across datasets.

The study further revealed that ArcFace supervision did not universally improve performance across architectures and datasets. Instead, the effectiveness of margin-based supervision depended on architectural design, dataset characteristics, and evaluation context, with fold-level analyses revealing consistent supervision patterns for several architectures. Similarly, the proposed Hybrid architecture achieved competitive identification and verification performance across both datasets, although these gains were accompanied by increased computational complexity.

The observed changes in model ranking and supervision behavior between LFW and FAGEv2 highlight the importance of multi-dataset benchmarking and subject-disjoint evaluation protocols when assessing face recognition systems. In addition, the fold-level analyses demonstrated the value of evaluating performance consistency alongside conventional average performance metrics.

Collectively, these findings support Hypotheses H1–H4 and emphasize the importance of reproducible evaluation, supervision sensitivity analysis, and multi-dataset benchmarking in contemporary face recognition research. The results further suggest that reliable biometric evaluation requires consideration of performance stability, computational trade-offs, and evaluation context in addition to absolute benchmark accuracy.

## 6 Conclusion

This study investigated the statistical reliability, cross-dataset behavior, and supervision sensitivity of convolutional, transformer-based, and hybrid face recognition architectures under subject-disjoint evaluation. A Hybrid multi-branch framework integrating ResNet50, MobileNetV3, and DeiT-Small feature extraction branches was evaluated under both Softmax and ArcFace supervision using five-fold subject-disjoint cross-validation on the LFW and FAGEv2 datasets.

The experimental findings demonstrated that comparative model superiority is strongly influenced by dataset characteristics, supervision strategy, and evaluation conditions. Although ArcFace supervision improved performance for certain architectures, it did not consistently outperform conventional Softmax optimization across all models and datasets. The results further revealed that the effectiveness of supervision strategies depends on both architectural design and dataset characteristics.

The proposed Hybrid architecture achieved competitive identification and verification performance across both datasets, demonstrating the potential benefits of combining complementary feature extraction paradigms within a unified framework. However, the experiments also revealed notable changes in model ranking and supervision behavior across datasets, highlighting the importance of evaluating face recognition systems under multiple benchmark conditions.

A key contribution of this study is the incorporation of fold-level supervision sensitivity analysis within a subject-disjoint evaluation framework. The combination of fold-level variance analysis, Wilcoxon signed-rank testing, fold consistency assessment, and multi-dataset benchmarking provided a more comprehensive evaluation of model behavior than average performance metrics alone.

Overall, the findings demonstrate that reliable face recognition benchmarking requires consideration of performance stability, supervision sensitivity, computational trade-offs, and evaluation context in addition to absolute benchmark accuracy. The study therefore contributes both a comparative evaluation of contemporary face recognition architectures and a reproducible benchmarking framework for assessing model behavior across heterogeneous evaluation settings.

## 7 Limitations and future work

Several limitations should be acknowledged.

First, the LFW evaluation was conducted on a filtered subset containing identities with at least 50 images to ensure sufficient intra-class representation under subject-disjoint cross-validation. Although this filtering strategy improves fold stability and enables more reliable estimation of performance variability, it alters the original identity distribution of LFW and may reduce the representativeness of unconstrained real-world deployment conditions.

Second, although fold-level variance analysis and Wilcoxon signed-rank testing were used to assess comparative performance, the five-fold cross-validation design provides a limited number of observations for statistical inference. Consequently, the statistical results should be interpreted as indicators of comparative consistency across folds rather than definitive evidence of population-level differences. Future work could investigate larger-scale repeated cross-validation protocols and alternative non-parametric statistical procedures to strengthen inference robustness.

Third, although the proposed framework demonstrated competitive performance across evaluation settings, both datasets remain moderate in scale compared with modern industrial face recognition benchmarks. Larger and more demographically diverse datasets may exhibit different supervision sensitivity, ranking behavior, and generalization characteristics.

Fourth, the study employed limited augmentation to preserve controlled evaluation conditions and reduce augmentation-induced variability during subject-disjoint benchmarking. Although this improves methodological consistency, stronger augmentation strategies may improve generalization performance, particularly for transformer-based and margin-supervised architectures.

Fifth, the proposed Hybrid architecture introduces increased computational complexity relative to standalone architectures. Although the model achieved strong identification and verification performance, more efficient fusion strategies such as attention-based weighting, adaptive feature gating, or dynamic branch selection may improve the complexity–performance trade-off.

Sixth, the experiments revealed that ArcFace supervision exhibits architecture-dependent and dataset-dependent behavior. Additional investigation is therefore required to better understand how angular-margin constraints interact with transformer representations, hybrid fusion mechanisms, and different dataset characteristics. Exploring alternative margin values, scaling parameters, and adaptive margin scheduling strategies may provide further insight into supervision sensitivity.

Seventh, the computational evaluation focused primarily on GFLOPs and parameter count. Future studies should additionally investigate inference latency, memory footprint, energy consumption, and hardware-specific deployment efficiency under real-world operating conditions.

The primary objective of this study was benchmarking and evaluation stability analysis under subject-disjoint protocols rather than qualitative interpretability analysis. Consequently, the experiments emphasized fold-level consistency, supervision sensitivity, and multi-dataset evaluation over visualization-based inspection of individual recognition outcomes.

Although the study focused primarily on quantitative benchmarking, future work may incorporate qualitative analysis of recognition outcomes, including representative examples of true positives, true negatives, false acceptances, and false rejections. Such analysis could provide additional insight into failure modes, embedding behavior, and dataset-specific error characteristics across architectures and supervision strategies.

Future research directions include evaluating larger-scale biometric benchmarks, investigating adaptive margin scheduling strategies, exploring more computationally efficient fusion mechanisms, and studying domain generalization approaches for improved performance across diverse evaluation settings. Additional work on fairness-aware evaluation, demographic bias analysis, and deployment-oriented optimization may further strengthen the practical applicability of face recognition systems.

In summary, this study demonstrates that reliable biometric benchmarking requires not only strong architectures but also reproducible evaluation protocols, multi-dataset benchmarking, fold-level analysis, and careful consideration of supervision sensitivity. The findings reinforce the importance of reproducibility, transparency, and evaluation consistency in contemporary face recognition research.

## Data Availability

The original contributions presented in the study are included in the article/supplementary material, further inquiries can be directed to the corresponding author.
